# A Review on the Extraction, Structural Analysis, and Antitumor Mechanisms of *Sanghuangporus* Polysaccharides

**DOI:** 10.3390/foods14040707

**Published:** 2025-02-19

**Authors:** Huaiyin Liang, Yanrui Ma, Yan Zhao, Nageena Qayyum, Fatao He, Jiewei Tian, Xiyun Sun, Bin Li, Yuehua Wang, Maoyu Wu, Guangpeng Liu

**Affiliations:** 1 College of Food, Shenyang Agricultural University, Shenyang 110866, China; 18735995024@163.com (H.L.); sun_xiyun@163.com (X.S.); libinsyau@163.com (B.L.); 2 Jinan Fruit Research Institute, China Federation of Supply and Marketing Co-Operatives, Jinan 250200, China; myr0223@163.com (Y.M.); ctcf13011717715@126.com (Y.Z.); fataohe@163.com (F.H.); tjw0824@126.com (J.T.); wmy-72@tom.com (M.W.); 3 College of Food Science and Engineering, Northwest A&F University, Xianyang 712100, China; nageena@nwafu.edu.cn

**Keywords:** *Sanghuangporus* polysaccharide, extraction, structure, tumor, mechanism

## Abstract

In recent years, the bioactive compounds extracted from *Sanghuangporus*, especially polysaccharides, phenols, and triterpenoids, have attracted great interest from people due to their extensive biological activity. Among them, polysaccharides are mainly extracted from the seed bodies, mycelium, and fermentation broth of *Sanghuangyuan*, exhibiting notable effects including immunomodulation, antitumor properties, and hypoglycemic effects. This article provides a comprehensive review of the extraction process, structural characteristics, and antitumor mechanism of *Sanghuangyuan* polysaccharides. First, the different extraction methods, such as hot water extraction, enzyme-assisted extraction, and ultrasonic-assisted extraction, are summarized. Then, the structure of the *Sanghuangporus* polysaccharide is studied in detail. Moreover, the antitumor mechanisms demonstrate significant inhibitory impacts on various malignant tumors, spanning gastric, hepatic, colorectal, breast, and prostate cancers. This groundbreaking revelation is of great significance for both the food and pharmaceutical sectors, presenting innovative pathways for *Sanghuangyuan* utilization and potentially inducing advancements in product development, treatment modalities, and therapeutic interventions.

## 1. Introduction

**Figure 1:**
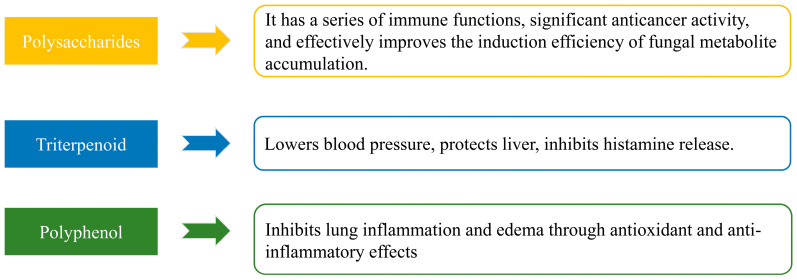
Diagram of the bioactive ingredients of *Sanghuangporus* and their function.

*Sanghuangporus* is a traditional edible and medicinal fungus that has been widely used for centuries to treat various ailments, such as diarrhea, menstrual disorders, stomach pain, and other conditions. Extensive traditional use and modern research have demonstrated that *Sanghuangporus* possesses multiple health benefits, including detoxification, improvement of gastrointestinal digestion and absorption, and potential life-prolonging effects. Chemical analysis has revealed that *Sanghuangporus* is rich in bioactive compounds, such as polysaccharides, flavonoids, triterpenoids, and phenolic compounds. These components have been shown to exhibit a wide range of biological activities, including antitumor, anticancer, hypoglycemic, hypolipidemic, and antioxidant properties. Collectively, these bioactive components contribute to the overall health-promoting effects of *Sanghuangporus*. In Figure 1, the bioactive components derived from *Sanghuangporus* and their associated health benefits are summarized, highlighting the multifaceted therapeutic potential of this traditional fungus.

To conduct a comprehensive literature review on the extraction, structural analysis, and antitumor mechanism of *Sanghuangporus* polysaccharides, we selected several major academic databases, including PubMed and Web of Science. These databases were chosen based on their broad coverage and authority in the field of food science. PubMed is renowned for its comprehensive collection of biomedical and life sciences literature, providing access to a vast array of peer-reviewed journals and research articles. It is an essential resource for identifying high-quality studies on the pharmacological effects of natural products, including *Sanghuangporus* polysaccharides. Web of Science offers a broad interdisciplinary scope and high-quality indexing, ensuring the inclusion of influential and widely cited research across various fields. It is particularly valuable for tracing the impact and citations of seminal studies on the antitumor mechanisms of *Sanghuangporus* polysaccharides [1]. These databases were selected to ensure that our literature search would be comprehensive, capturing a wide range of studies related to the antitumor mechanisms of *Sanghuangporus* polysaccharides. By utilizing these resources, we aim to provide a thorough and rigorous review of the current state of research in this area.

**Figure 2:**
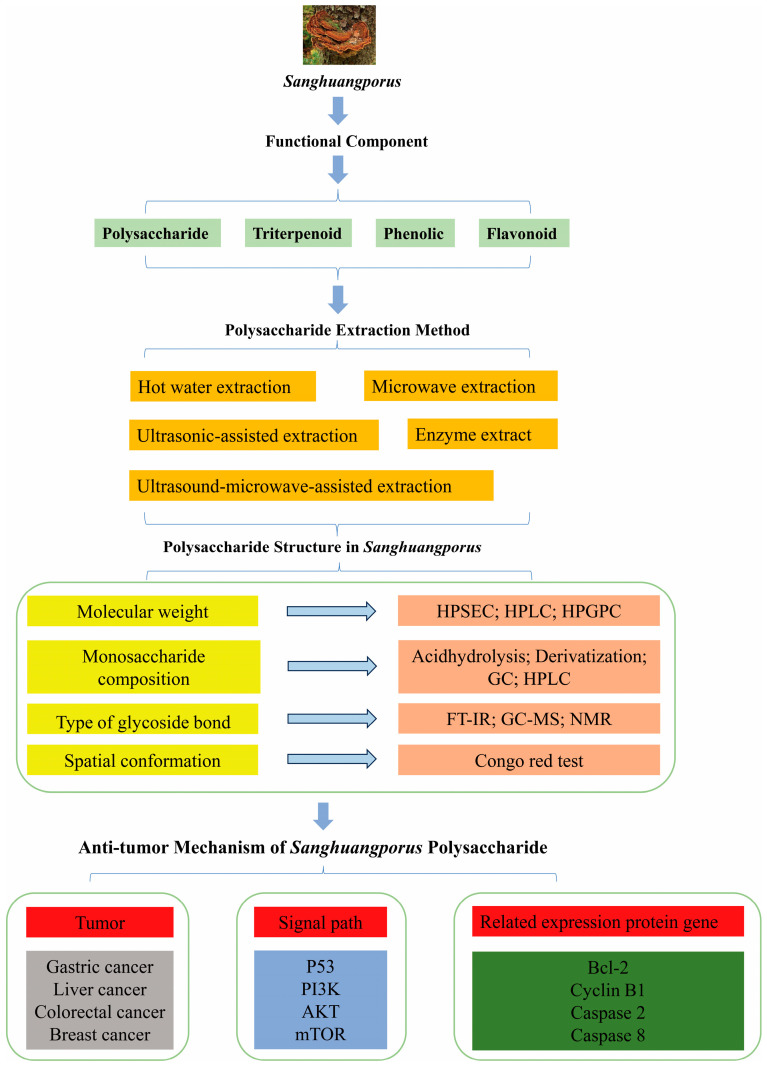
Extraction, structural characteristics, and antitumor mechanism of polysaccharides isolated from *Sanghuangporus*.

*Sanghuangporus* polysaccharides are macromolecules composed of long, complex chains of aldoses or ketoses linked by glycosidic bonds, which represent the largest proportion of the bioactive constituents of *Sanghuangporus*. These polysaccharides represent the largest proportion of the bioactive constituents in *Sanghuangporus.* They are involved in a multitude of biological processes, and provide a diverse array of nutrients and medicinal benefits [2]. As illustrated in Figure 2, the extraction, structural characterization, and antitumor activity of *Sanghuangporus* polysaccharides are key areas of research. Numerous studies have documented the nutritional value and pharmacological effects of *Sanghuangporus* polysaccharides. These polysaccharides not only provide energy to living organisms but also participate in the composition of the body. They have attracted widespread attention due to their bioactivity and health benefits.

*Sanghuangporus* polysaccharides have been demonstrated to possess promising immunomodulatory, antitumor, and antiangiogenic properties. These properties collectively result in the inhibition of tumor growth and the improvement of the efficacy of conventional cancer treatments. Recent studies have demonstrated that *Sanghuangporus* polysaccharide exert inhibitory effects on the proliferation and colony formation of SW480 human colon cancer cells. This inhibition is associated with decreased expression of the anti-apoptotic protein Bcl-2, increased release of cytochrome c from mitochondria, and reduced expression of the cell cycle protein cyclin B1 [3]. Ming-Ming Liu conducted a comprehensive investigation into the effects of *Sanghuangporus* polysaccharides on cell proliferation and cell cycle progression. The findings revealed that *Sanghuangporus* polysaccharides can inhibit the proliferation of human cervical cancer HeLa cells and significantly suppress the growth of gastric cancer SGC-7901 cells. Additionally, these polysaccharides may promote the division of RAW264.7 macrophage cells in the S phase of the cell cycle. A growing body of evidence suggests that the antitumor mechanisms of *Sanghuangporus* polysaccharides are multifaceted. They include cell cycle blockade, induction of apoptosis, antiangiogenesis, and the direct activation of various immune cells. These mechanisms contribute to their overall antitumor effects, highlighting their potential as a complementary therapy in cancer treatment.

In recent years, the incidence of various chronic diseases as well as cancer has been gradually increasing in recent years, and *Sanghuangporus* has a good inhibitory effect on many diseases. It has been found that *Sanghuangporus* polysaccharides can stimulate various components of the immune system, inhibit angiogenesis, interfere with key signaling pathways involved in tumor progression, and help the body to better identify and inhibit the growth of cancer cells by enhancing immune function. Currently, *Sanghuangporus* polysaccharides have been proven to possess good anti-inflammatory and antioxidant properties, among other biological activities. However, the research on the antitumor mechanisms of *Sanghuangporus* polysaccharides still lacks a comprehensive basis. Therefore, this paper systematically summarizes the research on *Sanghuangporus* polysaccharides in recent years, including the extraction methods, structural characteristics and antitumor mechanism of *Sanghuangporus* polysaccharides, to help people quickly understand the latest research progress of *Sanghuangporus* polysaccharides in the field of antitumor mechanisms, so that *Sanghuangporus* polysaccharides can maximally serve mankind, and to provide valuable insights for the next step of research on *Sanghuangporus* polysaccharides.

The full medicinal potential of *Sanghuangporus* has not been fully realized due to insufficient understanding of its systematics and biology. In the future, researchers can collaborate to explore the beneficial functions of *Sanghuangporus* through theoretical, practical, and procedural approaches. This collaborative effort will not only enable *Sanghuangporus* to maximize its contributions to human health but also serve as a valuable reference for the development and utilization of other edible fungi. In recent years, research on extracting polysaccharide components from *Sanghuangporus* has gained significant attention. However, there is still a lack of sufficient groundwork in studying the antitumor mechanisms of *Sanghuangporus* polysaccharides. To address this gap, this paper provides a systematic summary of research on *Sanghuangporus* polysaccharides. The paper presents a summary of extraction methods, structural characteristics, and the antitumor mechanism of *Sanghuangporus* polysaccharides. Additionally, it highlights the future development trends for *Sanghuangporus* polysaccharides to direct further research on polysaccharides from *Sanghuangporus* and other edible fungi.

## 2. Extraction of Polysaccharides from *Sanghuangporus*

Polysaccharides are bioactive components with promising medicinal potential, capable of exerting immunomodulatory, anticancer, and hypoglycemic effects [4]. Recent studies have shown that *Sanghuangporus* extracts possess a wide range of biological activities, including antitumor, antioxidant, antibacterial, antiviral, immunomodulatory, and neuroprotective activities. To extract polysaccharides effectively from *Sanghuangporus*, several pretreatment steps are necessary for the seed bodies or mycelium. Before polysaccharide extraction, these steps include crushing the seed bodies, degreasing with organic solvents, and other methods to eliminate impurities. Traditional methods for extracting polysaccharides from *Sanghuangporus* include hot water extraction (HWE) and the dilute alkali method. The composition and content of *Sanghuangporus* polysaccharides vary depending on the extraction method used, which, in turn, can affect their functional activity. HWE is frequently used technique due to its simplicity and low equipment requirements. However, it is characterized by prolonged extraction times and relatively low efficiency. The dilute alkali method can enhance extraction efficiency, but the addition of dilute alkali solution may potentially damage the structure of *Sanghuangporus* polysaccharides. Traditional methods for polysaccharide extraction are characterized by prolonged extraction times, low efficiency, and limited applicability. To address the limitations of traditional extraction methods, several innovative techniques have been developed for extracting polysaccharides from *Sanghuangporus*. These methods include microwave-assisted extraction (MAE), ultrasonic-assisted extraction (UAE), ultrasonic–microwave-assisted extraction (UMAE), pulsed electric field-assisted extraction (PEFAE), enzyme-assisted extraction (EAE), aqueous amphoteric extraction (ATPE), and other innovative extraction techniques [5]. These emerging techniques offer advantages such as shorter extraction times, higher yields, and better preservation of the bioactive structure of polysaccharides. For example, MAE and UAE can significantly reduce extraction time while maintaining or even enhancing the extraction efficiency. Enzyme-assisted extraction (EAE) can selectively break down cell walls, improving the release of polysaccharides without damaging their structure. Pulsed electric field-assisted extraction (PEFAE) can disrupt cell membranes, facilitating the release of intracellular components. In summary, the extraction of bioactive polysaccharides from *Sanghuangporus* is a crucial step in developing its medicinal applications. While traditional methods such as HWE and the dilute alkali method have been widely used, they are limited by long extraction times and potential structural damage to the polysaccharides. Emerging techniques like MAE, UAE, UMAE, PEFAE, EAE, and ATPE offer significant improvements in extraction efficiency and preservation of bioactivity. Future research should focus on optimizing these emerging techniques and exploring their potential for large-scale industrial applications.

**Figure 3:**
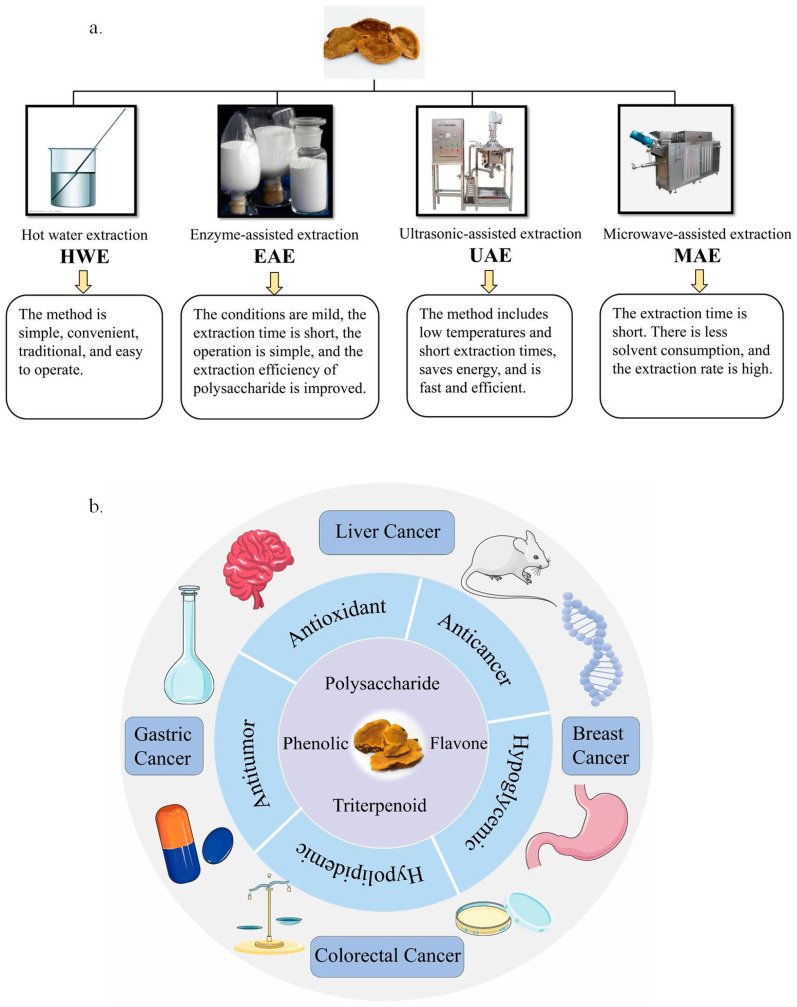
(**a**) Extraction methods and characteristics of polysaccharides from *Sanghuangporus*. (**b**) Biological activity diagram of *Sanghuangporus* polysaccharide.

This paper will provide detailed introductions to five polysaccharide extraction methods: HWE, EAE, UAE, MAE, and UMAE. The scope of application, advantages, and disadvantages of these innovative techniques will be elaborated upon, enabling individuals to select the optimal extraction method for achieving high efficiency and shorter extraction times. Figure 3a illustrates the extraction methods and characteristics of polysaccharides from *Sanghuangporus*.

### 2.1. Extraction Method of Polysaccharides from Sanghuangporus

#### 2.1.1. Hot Water Extraction (HWE)

Polysaccharides are large, polar compounds that readily dissolve in water, especially hot water. Hence, the HWE technique is widely employed for extracting polysaccharides. This method involves using high-temperature water to extract water-soluble polysaccharides from *Sanghuangporus*. Typically, the high-temperature treatment lasts for approximately 1.5 to 5 h, effectively removing insoluble substances and serving as a prevalent method for polysaccharide extraction.

In general, varying raw materials exhibit different extraction rates depending on factors such as solid–liquid ratios, extraction temperatures, and extraction times. This suggests that the optimal extraction conditions may vary depending on the specific raw materials utilized [6]. The HWE method is characterized by its simplicity, affordability, and minimal equipment requirements, making it suitable for industrial applications. However, it also has drawbacks, such as its time-consuming nature and the requirement for high extraction temperatures. Increased extraction temperatures and prolonged extraction times can potentially harm the structure of edible mushroom polysaccharides, leading to a decrease in their biological activity and degradation of essential chemical compounds. Additionally, the extraction process becomes more complex, limiting its development. The polysaccharide yield from HWE can be significantly influenced by various factors, including extraction time, extraction temperature, material–liquid ratio, etc. The effects of temperature, material–liquid ratio, extraction time, and ethanol concentration on the polysaccharide extraction rate were verified through both single-factor experiments and orthogonal experiments. The optimal extraction conditions were determined as follows: the ratio of water to *Sanghuangporus* mycelium was 1:40, with a temperature of 90 °C and an extraction time of 1.5 h, using an ethanol concentration of 80% (*v*/*v*). This concentration yielded the highest polysaccharide yield of 12.06% [7].

Jinze Liu conducted a single-factor experiment to optimize the extraction method for *Sanghuangporus* polysaccharides. The results indicated that the optimal extraction conditions were as follows: extraction temperature of 80 °C, extraction time of 3 h, and material–liquid ratio of 1:30 g/mL. Under these conditions, the polysaccharide yield of the extraction was 0.83 ± 0.12% [8].

Chao Wang employed HWE to isolate water chestnut polysaccharides, achieving an extraction rate of 12.0%. The study indicated that while the HWE did not notably affect the type of monosaccharides, it could alter their content and ratio. Furthermore, the polysaccharides extracted using hot water demonstrated an increase in inhibiting α-glucosidase activity in a dose-dependent manner [9]. Using HWE alone for polysaccharide extraction is not optimal. Studies have shown that employing high-pressure pretreatment of samples before HWE can significantly alter the relative abundance of total crude polysaccharide content [10]. The application of HHP in conjunction with hot water treatment led to notable alterations in the relative abundance of polysaccharides and proteins compared to using hot water treatment. Additionally, the combination of HHP and hot water treatment seemed to exhibit a synergistic effect on the production of soluble fractions [11]. Studies have indicated that increasing the extraction temperature from 60 °C to 90 °C results in higher crude polysaccharide production. This could be attributed to the higher temperature increasing the probability of cell wall rupture, leading to the release of more polysaccharides.

As mentioned above, the polysaccharides from *Sanghuangporus* can be extracted using hot water extraction (HWE). However, prolonged exposure to high temperatures or excessively high temperatures may reduce the biological activity and yield of the polysaccharides. In contrast, combining high-pressure pretreatment with hot water extraction can significantly increase the yield of polysaccharides.

#### 2.1.2. Enzyme-Assisted Extraction (EAE)

The EAE method is employed to extract polysaccharides from *Sanghuangporus* by promoting the decomposition of its cell wall, facilitating the release of polysaccharides. Enzymes effectively catalyze the hydrolysis and degradation of the fungal cell wall matrix, enabling the release of bioactive compounds trapped within the cell. This process facilitates the release of intracellular products into the extraction solvent, resulting in an accelerated release of intracellular biomolecules and higher polysaccharide yields. Generally used enzymes for solubilizing the cell wall include cellulase, hemicellulase, pectinase, protease, etc. [12]. EAE is preferred for polysaccharide extraction due to its moderate conditions, efficient extraction rate, minimal energy consumption, high yield, and straightforward and eco-friendly operation. Additionally, EAE is hindered by its higher cost, and the activity of enzymes may be affected by various factors such as temperature, oxygen levels, and more, making the extraction of *Sanghuangporus* polysaccharides using this method challenging. Huandong Yang employed the EAE method to isolate polysaccharides from *Cordyceps chrysanthemi*. It was observed that using cellulase for extraction significantly reduced both the extraction temperature and time, resulting in improved extraction efficiency [12]. Cheng Junwen utilized a composite enzyme consisting of cellulase, pectinase, and protease in a ratio of 2:1:1 to enzymatically treat *Sanghuangporus* seeds. The ultrasonic synergistic composite enzyme extraction of *Sanghuangporus* polysaccharides was optimized, resulting in the following optimal conditions for polysaccharide extraction from *Sanghuangporus* seeds: ultrasonic power of 360.6 W, liquid/feed ratio of 32.5:1, and ultrasonic time of 32.7 min. These yielded a polysaccharide rate of 3.31%. Yang Zhu optimized the EAE process of polysaccharides using a Box–Behnken design of experiments. Several potential mechanisms for antitumor activity were proposed. The results revealed that the polysaccharides from Monkey mushrooms primarily consisted of glucose, galactose, mannose, caramel, arabinose, xylose, and rhamnose. Additionally, they exhibited a significant inhibitory effect on the growth of HeLa tumor cells [13]. Yong-ming Zhao extracted polysaccharides from shiitake mushrooms using both hot water extraction and enzyme-assisted extraction. The yield of polysaccharides obtained through hot water extraction was (10.68 ± 0.21)%, while EAE yielded (15.65 ± 0.18)%, indicating a significant increase in yield by 46.54% with EAE. The presence of the cell wall limits the extraction of polysaccharides from plants, making cell wall degradation a crucial step in releasing polysaccharides from shiitake mushrooms. This study demonstrated that EAE resulted in higher polysaccharide extraction, making it an effective, economical, sustainable, efficient, and cost-effective method [14]. EAE is a commonly used method to extract polysaccharides from *Sanghuangporus*, which can promote cell wall decomposition and increase polysaccharide yield. The commonly used enzymes include cellulase, hemicellulase, pectinase, and protease, but the enzyme activity is affected by temperature and other factors, so it is difficult to extract polysaccharides from *Sanghuangporus* using this method.

#### 2.1.3. Ultrasonic-Assisted Extraction (UAE)

Ultrasonic extraction of polysaccharides primarily relies on the high-frequency vibration of ultrasound, which induces high-speed movement of molecules within the sample. This leads to the rupture of the cell wall, a reduction in particle size, and rapid release of polysaccharides from the sample, thereby accelerating the extraction process and significantly enhancing polysaccharide extraction efficiency. However, it may also result in the degradation of some polysaccharides. The ultrasonic method operates based on acoustic cavitation (a mechanical effect), which can generate low-molecular-weight components tailored for specific purposes. This method ensures short and efficient extraction of polysaccharides, although it can also induce changes in the material structure of polysaccharides. Consequently, this may affect both the physicochemical properties and biological activity of *Sanghuangporus* polysaccharides. Therefore, in the process of extracting polysaccharides using ultrasonic waves, factors such as the frequency and intensity of ultrasonic waves must be strictly controlled [15].

Yang Zhang optimized the UAE of polysaccharides (PKFs) from *Kangxianhua* flowers using response surface methodology. The findings revealed that, with a liquid–solid ratio of 59:1 mL/g, an ultrasound power of 404 W, an extraction time of 48 min, and a temperature of 66 °C, the yield of PKFs reached 26.8 ± 1.76%. This yield was 2.6 times higher compared to that using hot water extraction. Different analyses indicated that the PKFs extracted through ultrasonication exhibited reduced molecular weights and enhanced antioxidant capacity compared to the polysaccharides obtained by other methods. In addition to enhancing the yield, UAE also elevated the glyoxylate content, total flavonoid content, and water solubility of polysaccharides [16]. Michelle Dorcas Mtetwa optimized the UAE of *Acanthus ilicifolius* polysaccharides through single-factor experiments and Box–Behnken design, achieving optimal extraction conditions with an ultrasonic power of 150 W. The optimal extraction conditions included a temperature of 53 °C, an extraction time of 50 min, and a liquid-to-feed ratio of 35 mL/g, resulting in the highest polysaccharide yield of 2.46%. Following analysis, it was discovered that polysaccharides extracted via ultrasound exhibited greater in vitro antioxidant activity compared to those extracted via hot water. This may be attributed to the lower molecular weights and higher phenolics and protein fractions in the ultrasonically extracted polysaccharides [17]. Additionally, they observed that UAE influenced the monosaccharide composition, ratio, and molecular weight range of *Volvariella volvacea* polysaccharides [18]. Jing-Kun Yan utilized ultrasound for the extraction of *Sanghuangporus* polysaccharides. The findings indicated that ultrasonic treatment decreased the molecular weight of *Sanghuangporus* polysaccharides while enhancing their antioxidant activity [19]. Jinze Liu also employed UAE for *Sanghuangporus* polysaccharides, with optimized parameters including an extraction temperature of 60 °C, extraction time of 60 h, material–liquid ratio of 40 g/mL, and ultrasonic power of 70 W. Under these conditions, the extraction rate was 1.41 ± 0.11%. Bin Neng Gao’s team optimized the ultrasonic-assisted extraction of polysaccharides and found that when the ultrasonication time exceeded 20 min, the yield of *Sanghuangporus* polysaccharides decreased. This decrease could be attributed to the cavitation effect of ultrasonication, which promotes the oxidative degradation of polysaccharides and increases the solubility of impurities, thus reducing the extraction rate of the polysaccharides. Additionally, Jing-Kun Yan employed ultrasound to extract *Sanghuangporus* polysaccharides, and the results indicated that ultrasonic treatment reduced the molecular weight of *Sanghuangporus* polysaccharides while enhancing their antioxidant activity. Additionally, excessively high ultrasonic extraction temperatures also resulted in polysaccharide degradation. When the temperature exceeded 60 °C, the extraction rate decreased significantly. This decline was attributed to the high temperature causing the complete rupture of the cell wall of *Sanghuangporus* polysaccharides. It was speculated that the polysaccharides of *Sanghuangporus* primarily consisted of glucose, mannose, and galactose, with these three monosaccharides accounting for 97.2% of the total sugar content. In comparison to traditional HWE, the UAE method effectively decreased the extraction temperature, shortened the extraction time, and improved the extraction rate of polysaccharides. Additionally, it enhanced the solubility of impurities, resulting in a reduced extraction rate of the polysaccharides. Moreover, excessively high ultrasonic extraction temperatures also resulted in polysaccharide degradation. When the temperature exceeded 60 °C, there was a notable decrease in the extraction rate. This decline was attributed to the elevated temperature causing the complete rupture of the cell wall of *Sanghuangporus* polysaccharides. It was speculated that the polysaccharides from *Sanghuangporus* predominantly comprised glucose, mannose, and galactose, with these three monosaccharides collectively accounting for 97.2% of the total sugar content. The UAE method, in contrast to traditional HWE, notably decreased the extraction temperature, shortened the extraction time, and enhanced the extraction rate [20].

UAE utilizes the high-frequency vibrations of ultrasonic waves to promote the rapid release of polysaccharide molecules from *Sanghuangporus*. This accelerates the extraction process and improves the extraction efficiency. However, it may also lead to degradation of the polysaccharides. Overall, UAE can reduce the extraction temperature, shorten the extraction time, and increase the extraction yield.

#### 2.1.4. Microwave Extraction (MAE)

MAE is a groundbreaking extraction system that enables the extraction of polysaccharides with high yield in a shorter duration and with decreased solvent and energy consumption. Because of its rapid heating, this extraction method provides higher extraction rates compared to other techniques like Soxhlet and supercritical fluid extraction. Furthermore, MAE, as a sample preparation technique, can be seamlessly integrated with analytical systems such as chromatography and spectroscopy to assess bioactive compounds. Hence, MAE is regarded as a promising method for extracting polysaccharides with notable bioactivity. Microwave irradiation, causing deep cell wall rupture in brief extraction durations, enhances the yield and bioactivity of polysaccharides by microwaves’ impact on both the solvent (volumetric heating) and the sample (enhanced release of polysaccharides from the matrix into the solvent). Several structural factors significantly influence the anti-free radical, antioxidant, and antimicrobial activities of polysaccharides extracted from biological sources. These factors include the composition of monosaccharides, glyoxylate content, molecular weight, and degree of esterification. Polysaccharides extracted from MAE exhibit outstanding biological properties attributed to the intact molecular structure containing functional glycosidic bonds, a high molecular weight, and glyoxylate content. MAE employs high-energy microwave penetration to transfer energy through the cell wall into the cytoplasm, elevating temperature and pressure within the cell. As pressure reaches a critical point, the cell wall expands and ruptures, facilitating the release of polysaccharides and other cellular substances, thereby notably enhancing the polysaccharide extraction efficiency. MAE significantly decreases solvent usage and reduces extraction time, allowing for better contact between the contents and the solvent, leading to a notable improvement in extraction efficiency [21].

In the food industry, the MAE of polysaccharides yields various effects. It alters the structural configuration of polysaccharides by loosening the cell wall matrix and separating thin-walled cells. This process decreases the molecular weight of extracted polysaccharides, thereby enhancing both the extraction rate and antioxidant activity. Furthermore, polysaccharides, extracted via MAE, exhibit enhanced antibacterial, antifungal, and antiviral effects, thereby significantly boosting their biological activity [22].

Wei-Cai Zeng employed MAE for extracting polysaccharides from *Auricularia auriculae* and investigated their antioxidant activity. The findings revealed that polysaccharides extracted using the microwave method exhibited low molecular weights yet demonstrated significant antioxidant capacity [23].

The notable increase in antioxidant activity observed is attributed to the low molecular weight of polysaccharides obtained via the microwave method. These biomolecules possess numerous free hydroxyl groups, which reduce viscosity and enhance compound solubility [24]. Polysaccharides extracted via MAE notably enhanced the reducing power (FARP) of trivalent iron ions. Okra polysaccharides extracted via MAE exhibited higher FARP at all concentrations compared to those obtained through HWE [25].

Therefore, MAE is an efficient polysaccharide extraction system. It can extract *Sanghuangporus* polysaccharides in a short time while reducing solvent and energy consumption. Additionally, polysaccharides extracted using MAE exhibit significant antioxidant, antibacterial, and antiviral activities.

#### 2.1.5. Ultrasound–Microwave-Assisted Extraction (UMAE)

When extracting polysaccharide components from *Sanghuangporus*, the extraction effectiveness of each method, whether UAE or MAE, is consistently limited. For instance, UAE consumes more energy and may introduce impurities, whereas MAE might result in uneven heating and limitations in mass transfer, potentially causing damage to the polysaccharide structure. In light of these limitations, researchers have integrated ultrasonic and microwave technologies and discovered that UMAE amalgamates the benefits of both methods, offering numerous advantages. This method has been employed for the extraction of various natural plant compounds, notably reducing extraction time, cutting costs, and enhancing the polysaccharide extraction rate.

MAE utilizes the conversion of high-frequency electromagnetic energy into thermal energy, resulting in a more efficient extraction process, albeit with uneven heat distribution. In contrast, ultrasonic extraction relies on cavitation phenomena for intense material mixing but does not generate high thermal energy levels. Hence, ultrasonic–microwave-assisted extraction (UMAE), employing the synergistic action of ultrasound and microwave, has been employed for polysaccharide extraction [26].

Yanlin Zhang examined the extraction of polysaccharides from *Dictyophora indusiata* (DPs) and found that various extraction methods did not significantly affect the types of glycosidic bonds and sugar rings, as well as the chemical composition, monosaccharide composition, absolute molecular weight (Mw), and molecular conformation. However, the UMAE treatment inflicted more damage to the cell wall, yielding higher amounts of extracted polysaccharides with increased antioxidant capacity. This outcome likely stemmed from the altered conformations, stretching, and reduced degradation of DPs in high-molecular-weight fractions due to the combined impact of microwaves and ultrasound [21].

Yiyong Chen employed UMAE for extracting polysaccharide components from birch mushrooms, observing significantly reduced extraction time compared to single-technique methods, completing the process in just 19 min. Additionally, the yield and purity of the polysaccharides were enhanced [27].

The study revealed that the yield of *Ganoderma lucidum* polysaccharides extracted via the UMAE method surpassed that of HWE by 115.56% and that of UAE by 27.7%. The researchers proposed that the mechanical synergistic effect of ultrasound and microwaves on the cell wall likely enhanced the efficiency of UMAE [28]. Polysaccharides extracted from Pleasant Tree fruits using UMAE exhibited significantly higher yields compared to those extracted using HWE, UAE, and MAE. This phenomenon may be attributed to the elevated pressure resulting from ultrasonic cavitation, causing cell wall disruption and enhancing polysaccharide solubility. Additionally, during the subsequent microwave extraction, the conversion of high-frequency electromagnetic energy into thermal energy facilitated the extraction process. Hence, the combined action of ultrasound and microwaves in UMAE results in increased efficiency [29].

UMAE is an extraction method that combines ultrasonic and microwave technology to isolate natural plant compounds, especially polysaccharides. UMAE has many advantages, which can shorten the extraction time, reduce the cost, and increase the extraction rate of polysaccharides; UMAE can improve the yield and purity of polysaccharides, cause greater damage to the cell wall, and extract higher polysaccharide content. Table 1 shows the effects of different extraction methods on the extraction yield and biological activity of polysaccharides from *Sanghuangporus* under different extraction conditions.

### 2.2. Purification of Polysaccharides from Sanghuangporus

After extraction, the crude *Sanghuangporus* polysaccharide is a mixture containing proteins, pigments, and other impurities. To obtain a single-component polysaccharide, further purification is required. Commonly used purification methods include ethanol precipitation, solvent extraction, hydrogen peroxide, adsorption, ion exchange column chromatography, and gel filtration chromatography, among others. Yan Wu used an aqueous two-phase system (ATPS) based on choline chloride ([Chol]Cl) and potassium hydrogen phosphate (K_2_HPO_4_) for the separation of polysaccharides and proteins in *Sanghuangporus*. The system was optimized with the following composition: 68.9% (*w*/*v*) K_2_HPO_4_, 20% (*v*/*v*) [Chol]Cl, 10 mg·mL^−1^ of aqueous crude extract, and 4 mL of distilled water. When the oscillation time was set to 30 min and the temperature was maintained at 21.2 °C, the polysaccharides obtained from this ATPS method exhibited significant antioxidant effects compared to those obtained using conventional purification methods [30].

**Table 1:** Effects of different extraction methods on the extraction yield and bioactivity of polysaccharides from Sanghuangporus under different extraction conditions.

Extraction Method	Extraction Condition	Yield	Biological Activity	References
Ultrasonic-assisted extraction	Temperature: 60 °C; Time: 60 min; Solid–liquid ratio: 1:40 g/mL; Ultrasonic power: 70 W.	1.41 ± 0.11%	PIP significantly inhibited the levels of pro-inflammatory cytokines, and promoted the level of the anti-inflammatory cytokine IL-10 in lipopolysaccharide (LPS)-induced RAW 264.7 cells.	[8]
Hot water extraction	Temperature: 80 °C; Time: 3 h; Solid–liquid ratio: 1:30 g/mL.	0.83 ± 0.12%	Antioxidant and anti-inflammatory.	[8]
Ultrasound-assisted extraction	Temperature: 60 °C; Time: 20 min.	12.98%	PIP has a stronger inhibitory ability for *S. aureus* and *E. coli* and a slightly weaker inhibitory effect for *B. subtilis*.	[20]
Phosphotungstic acid-assisted extraction	Temperature: 90 °C; Time: 2 h; 0.5% (*w*/*v*) of H3O40PW12·xH_2_O.	5.2%	PIP significantly promoted the abundance of Firmicutes and Actinobacteriota while demonstrating an antiproliferative effect against Proteobacteria and Bacteroidota.	[31]
Delignification method	Temperature: 70.3 °C; Ratio of water to raw material: 34.7 mL/g; Amount of acetic acid: 0.32 mL every time.	10.28%	Noticeable antioxidant activity and strong protection against oxidative DNA damage.	[32]
Ultrasound-assisted extraction	Temperature: 70 °C; Time: 34 min; Solid–liquid ratio of 1:40; Ultrasound power of 350 W.	51.06 ± 0.34%	Antioxidant activity, and reduce H_2_O_2_-induced oxidative damage to cells in vitro.	[33]
Microwave extraction	Microwave extraction for 15 min; three times.	5.37%	Antitumor and immune activities.	[34]
Enzyme pretreatment followed by ultrasonic before TPP	Temperature: 35 °C; Centrifugation: ×4500 rpm, 10 min.	25.40%	Antioxidant, hypoglycemic, hypouricemic, immunostimulatory, and antitumor activities.	[35]
Three-phase partitioning	Ammonium sulfate concentration: 20% (*w*/*v*); Ratio of cultured broth to t-butanol: 1.0:1.5 (*v*/*v*); Time: 30 min; Temperature: 35 °C.	52.09%	Radical-scavenging abilities, antioxidant capacities, α-amylase and α-glycosidase inhibitory activities, and macrophage stimulation activities.	[36]

Several studies have shown that ion exchange column chromatography and gel filtration chromatography are more effective methods for the purification of *Sanghuangporus* polysaccharides. Ion exchange chromatography uses charge interactions to separate *Sanghuangporus* polysaccharides with different acidity and alkalinity, while gel filtration chromatography uses gel molecular sieves with a network structure to separate *Sanghuangporus* polysaccharides with different molecular weights. For example, Jinze Liu combined DEAE-52 cellulose column chromatography with NaCl solution to elute the crude polysaccharides of *Sanghuangporus* by 40% (*v*/*v*) ethanol precipitation to obtain single-molecule polysaccharides [8]. Qing Ge used hot water extraction, DEAE-Sepharose fast flow anion exchange, and gel filtration chromatography to isolate and purify the seed bodies of *Sanghuangporus* to obtain water-soluble polysaccharides, which were purified as β-d glucan complexes with a molecular weight of 230 kDa [37]. Shi-Chao Li sequentially purified water-soluble intracellular polysaccharides from *Sanghuangporus* mycelium by ethanol fractionation and precipitation, ion exchange, and volumetric exclusion chromatography [38]. Chao Zhao optimized the extraction conditions of *Sanghuangporus* polysaccharides using orthogonal tests and purified the crude polysaccharides by DEAE Sephadex A-50 and Sephadex G-200 chromatography, and it was found that the purified polysaccharides were mainly a kind of branched chain polysaccharide with α-bonds and β-bonds, and had a pyranose ring conformation [7]. Jing-Kun Yan separated and purified the water-soluble polysaccharide (PLP1-I) from *Sanghuangporus* mycelium by hot water extraction, ethanol precipitation, and DEAE Sepharose Fast Flow and Sephacryl S-400 HR columns. The obtained PLP1-I was a neutral heteropolysaccharide consisting of glucose and galactose with an average molecular weight of ~290,000 kDa [19].

In summary, the isolation and purification of *Sanghuangporus* polysaccharides was carried out by crushing and drying *Sanghuangporus* seed bodies, followed by hot water extraction, ethanol precipitation, and then polysaccharide extraction and purification operations. *Sanghuangporus* polysaccharides exert their anticancer effects by inhibiting tumor cell growth, inducing apoptosis, regulating cell differentiation, enhancing the antitumor effects of other drugs, etc. Several cancer-related signaling pathways, such as PI3K-Akt, MAPK, and NF-κB, have been shown to be regulated by *Sanghuangporus* polysaccharides, which play a key role in the antitumor effects of *Sanghuangporus* polysaccharides. In the extraction of polysaccharides from *Sanghuangporus*, differences in extraction methods may affect the structure, monosaccharide composition, and glycosidic linkage type of *Sanghuangporus* polysaccharides, which are closely related to the biological activities of polysaccharides. Therefore, in order to increase the yield of polysaccharides, researchers have used solvents such as acids and bases, as well as ultrasonic and enzymatic methods to extract polysaccharides, and, in the future, more new extraction methods and improved traditional extraction methods will be used to extract polysaccharides from *Sanghuangporus* in order to increase the yield of polysaccharides and maintain their bioactivities.

## 3. Structure of Polysaccharides from *Sanghuangporus*

Polysaccharides, essential biological macromolecules derived from renewable resources such as plants, animals, and microorganisms, exhibit effectiveness in scavenging free radicals, boosting antioxidant enzyme activities, and inhibiting lipid peroxidation.

To date, more than 60 polysaccharides have been extracted from *Sanghuangporus*, but only a few have been structurally characterized. The biological activity of polysaccharides is closely related to their structural features, including molecular weight, monosaccharide composition, glycosidic bonding, degree of branching, and chain conformation, The antitumor activity of *Sanghuangporus* polysaccharides is primarily dependent on their fine structure at all levels. Previous studies have shown that the structure, glycosidic bonding, and chemical composition of polysaccharides are related to their ability to modulate the characteristics of the gut microbial community. Additionally, the biological activity of polysaccharides is also affected by their chemical composition, molecular weight, and conformation. In order to better understand the antitumor mechanism of *Sanghuangporus* polysaccharides, their structural features need to be studied in detail.

Therefore, there has been extensive research on the extraction and purification methods, structural characterization, and chemical modification of fungal polysaccharides, leading to a significant research foundation in this area [39]. Figure 3b illustrates the biological activity of polysaccharides from *Sanghuangporus*. As of now, over 60 polysaccharides have been extracted from *Sanghuangporus*, but only a few of them possess well-defined structural characteristics, typically determined by their molecular weight (Mw), monosaccharide composition (MC), glycosidic bonding type, and spatial conformation. The antitumor activity of *Sanghuangporus* polysaccharides primarily relies on their intricate structure across all levels [40]. Prior research has indicated that the structure, glycosidic bonding, and chemical composition of polysaccharides are linked to their capability to regulate the traits of the gut microbial community. Furthermore, the biological activity of polysaccharides is influenced by their chemical composition, molecular weight, and conformation. Detailed examination of its structural characteristics is necessary to provide a more comprehensive understanding of *Sanghuangporus’* antitumor mechanism. Presently, various advanced techniques are accessible for elucidating the fundamental structural features of fungal polysaccharides, including Fourier Transform Infrared Spectroscopy (FT-IR), Nuclear Magnetic Resonance (NMR), high-performance liquid chromatography (HPLC), gas chromatography–mass spectrometry (GC-MS), and other methodologies. Utilizing these advanced techniques for determining and analyzing *Sanghuangporus* polysaccharides yields valuable data for investigating their antitumor mechanism. This holds great significance for studying the anticancer effects of fungal polysaccharides.

### 3.1. Molecular Weight

The molecular weight is a significant parameter that indicates the chemical properties of polysaccharides. In the *Sanghuangporus* genus, the molecular weight ranges from 1.00 × 10^3^ Da to 1.00 × 10^7^ Da [41]. High-performance volumetric exclusion chromatography (HPSEC), high-performance liquid chromatography (HPLC), and high-performance gel permeation chromatography (HPGPC) are frequently employed for determining the molecular weight of *Sanghuangporus* polysaccharides [42]. In 2003, Jin Yong conducted research on the structure of 10 polysaccharides isolated from *Sanghuangporus* mycelium.

It was found that samples from wild *Sanghuangporus* strains grown in maize medium exhibited a notable inhibitory effect on tumor cell growth. It was hypothesized that the variance in antitumor activity among different polysaccharides could be attributed to differences in molecular weight [43]. Through the characterization and comparison of the chemical structures of five mushroom polysaccharides, Wang Kaiping found a correlation: as the molecular weight of the polysaccharides increased, the degree of branching (DB value) decreased, water solubility increased, and the antitumor effect strengthened. Thus, the DB value could be significant in assessing the antitumor effect [44]. Certainly, variations in experimental raw materials and methods could result in significant differences in molecular weight. Therefore, it was imperative to strive for consistency in experimental conditions during molecular weight determination to minimize inconsistencies.

### 3.2. Monosaccharide Composition

The composition of monosaccharides in *Sanghuangporus* polysaccharides is determined through acid hydrolysis and derivatization, followed by analysis using gas chromatography (GC), high-performance liquid chromatography (HPLC), and gas chromatography–mass spectrometry (GC-MS) [45]. Polysaccharides extracted from *Sanghuangporus baumii* have been isolated into two fractions: SPB and SPC. Both fractions primarily consist of glucose, galactose, and mannose, with residues mainly interlocked in a pattern of (1 → 6)-galp-.

It was shown that *Sanghuangporus* polysaccharides primarily consisted of galactose, glucose, and mannose in varying molar ratios. Furthermore, the presence of additional monosaccharides like arabinose and rhamnose was detected in *Sanghuangporus* polysaccharides. Deng Junwen conducted research on the polysaccharide fraction (SSIPS1) derived from *Sanghuangporus* mycelium. The findings indicated that SSIPS1 primarily consists of glucose (94.8%) with minor quantities of galactose (5.2%). Furthermore, distinct cultivation techniques resulted in notable variations in the monosaccharide composition and the molar ratio of polysaccharide fractions isolated from *Sanghuangporus* [2]. Xiaopeng Liu et al., used the internal standard method to calculate the composition of monosaccharides, and the results showed that the polysaccharides of Sanghuangporus were mainly composed of 51.9% glucose and 18.9% galactose, with small amounts of fucose, mannose, and glucuronic acid.

Wang Qiong conducted a comprehensive review of the monosaccharide composition and molar ratio of polysaccharides derived from various *Ganoderma* species over several decades. They observed that polysaccharides obtained from the same strain but from different sources, such as substrate, mycelium, or fermentation broth, exhibit diverse biological activities based on their monosaccharide compositions. Additionally, their research indicated that enzymes involved in fungal polysaccharide synthesis, such as phosphoglucose isomerase, α-phosphoglucose mutase, and UDP-Glc pyrophosphorylase, play a crucial role in regulating polysaccharide production and monosaccharide composition [46].

### 3.3. Type of Glycoside Bond

To date, there has been minimal exploration into the glycosidic bond types of *Sanghuangporus* polysaccharides. Yuan Qingxia investigated the antioxidant and antitumor properties of these polysaccharides. The polysaccharides were subjected to methylation, hydrolysis, reduction, and conversion into partially methylated glycol acetates (PMAAs). The glycosidic bond types were subsequently determined through GC-MS analysis [47]. Research has shown that the presence of glyoxylate residues in polysaccharides can influence their properties and modify the solubility of associated polysaccharide linkages. Consequently, Gong Pin quantified the glyoxylate content in apricot mushroom polysaccharides. The findings revealed that apricot mushroom polysaccharides contain minimal amounts of glyoxylate, suggesting they may be classified as neutral polysaccharides [48]. Jing-Kun Yan utilized NMR spectroscopy to elucidate the structure of *Sanghuangporus* polysaccharides. It was revealed that the polysaccharide PLP1-I from *Sanghuangporus* contained α-configured pyranose units and was confirmed to contain dextran with α-glycosidic bonds. Through a combination of methylation analysis, GC, GC-MS, FT-IR, and NMR, it was determined that PLP1-I consists of (1 → 4)-α-d-glucopyranosyl residues and (1 → 3,6)-α-d-galactopyranosyl residues in the main chain, with branching occurring at O-6. The branch comprises (1 → 3)-α-D-galactopyranosyl residues terminated by (1→)-α-D-glucopyranosyl residue. The polysaccharide structure derived from *Sanghuangporus* in this investigation differs from those previously documented from other *Sanghuangporus* samples, likely due to variations in the source of raw materials [19]. Liu et al. analyzed the glycosidic bonds of polysaccharides using GC-MS and found that the polysaccharides of Sanghuangporus were mainly linked by five major sugar residues, of which →6-Glcp-1→ was the most abundant, accounting for 63.35%.

Furthermore, alterations in glycosidic bonding can impact molecular weight. Mengen Yu explored the shifts in polysaccharide molecular weight and observed a notable increase in reducing sugar content after gastrointestinal digestion. This suggests that the cleavage of glycosidic bonds resulted in a decrease in molecular weight [49].

### 3.4. Spatial Conformation

The molecular weight and chain conformation are pivotal structural characteristics of polysaccharides. The conformation of polysaccharides primarily depends on the chain structure and intramolecular forces, such as hydrogen bonding, dipolar interactions, hydrophobic forces, and electrostatic forces. Nonetheless, external factors such as heat treatment, ultrasound, pH, ions, or solvents can induce changes in the conformation of polysaccharides in solution [1]. The biological effectiveness of mushroom polysaccharides is widely recognized to be significantly influenced by their chain conformation and molecular weight [50]. The Congo red test is commonly employed to assess the spatial conformation of polysaccharides. Polysaccharides with a triple-helical conformation can interact with Congo red, forming complexes known as Congo red–polysaccharide complexes. This interaction results in a redshift in the maximum absorbed wavelength (λmax). When the triple-helical conformation is disrupted by strong bases, a reduction in the red-shift of the Congo red-polysaccharide complexes can be observed. Yu Hua investigated alterations in the triple-helical structure of shiitake mushroom polysaccharides by measuring λmax before and after ultrasonic treatment. The study revealed significant variations in λmax values depending on the different ultrasonic frequency modes applied.

At frequencies of 60 kHz, 40/60 kHz, and 20/40/60 kHz, no notable redshift was observed post ultrasonication, suggesting that the triple-helical structure within polysaccharides was disrupted. This disruption is likely attributed to the effects of ultrasonication, including cavitation and mechanical forces, which interfere with the inter- and intramolecular hydrogen bonding responsible for maintaining the triple-helical structure [51].

Yi-Ching Cheung investigated the impact of ultrasonic degradation on the conformation of polysaccharide chains in curd polysaccharides within alkaline solutions. The study revealed a notable decrease in viscosity with higher ultrasonic power and prolonged ultrasonication time. Additionally, the conformation of the curdlan chain shifted from a relatively rigid triple helix to a more flexible single helix and random coil. Direct evidence of this conformational change was provided by the Congo red test [52].

Surenjav et al. determined that the physiological effectiveness of polysaccharides fluctuates based on structural characteristics such as molecular weight, degree of branching, and spatial conformation. They highlighted that the presence of a β-(1 → 3)-Glcp backbone with branching at the O-6 site was significant for its antitumor activity [53]. Hence, the spatial conformation plays a vital role in the antitumor efficacy of polysaccharides from *Sanghuangporus*.

## 4. Antitumor Mechanism of *Sanghuangporus* Polysaccharide

### 4.1. Current Status of Cancer Research

A tumor refers to the uncontrollable proliferation of human cells, characterized by abnormal and unregulated division and growth. A malignant tumor, known as cancer, exhibits rapid proliferation and is not constrained by geographical or spatial boundaries. Currently, the incidence of malignant tumors is rapidly increasing, making cancer the second most prevalent cause of death globally. Cancer is a set of diseases distinguished by unregulated cell proliferation, a propensity to invade neighboring tissues, and the transformation of normal cells into cancerous ones.

While the precise cause of cancer development remains incompletely understood, ongoing research indicates that factors such as aging, exposure to chemicals and radiation, infections, poor dietary habits, family history, and obesity may contribute to its onset [54]. Cancer has emerged as a significant challenge for humanity, prompting countries worldwide to actively implement measures aimed at reducing the occurrence of tumors.

**Figure 4:**
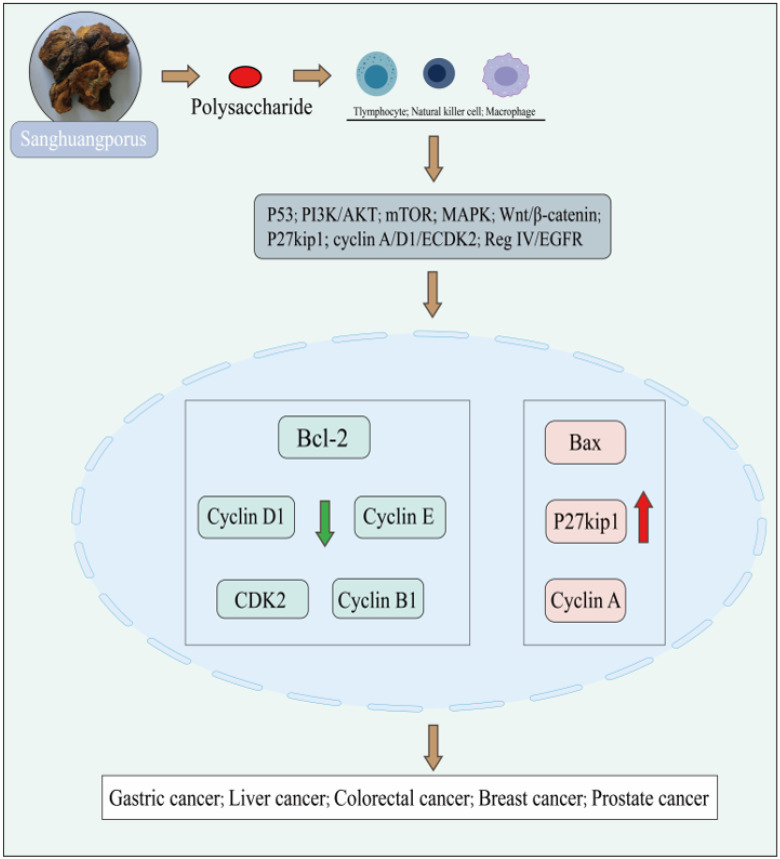
Schematic diagram of molecular mechanism of antitumor activity of polysaccharide isolated from *Sanghuangporus*. (Downward green arrows indicate down-regulated genes and upward red arrows indicate up-regulated genes).

Until now, an increasing number of studies have revealed that edible medicinal fungi can serve as effective treatments for cancer patients. Recent research has demonstrated that polysaccharides, bioactive compounds derived from medicinal and edible fungi, can exhibit anticancer effects through the activation of T lymphocytes, macrophages, and NK cells. It has been proposed that polysaccharides could potentially hinder tumor cell growth by elevating antioxidant levels and scavenging ROS free radicals within cancer cells. In contrast, the polysaccharides found in the fruiting bodies of *Sanghuangporus* consist of β-glucan polymers, which are regarded as potential anticancer agents against various cancer cell lines, such as those associated with prostate, gastric, liver, colon, and breast cancers. Their anticancer effects and mechanisms primarily involve the inhibition of cancer cell proliferation, disruption of the cell cycle, and regulation of apoptosis. Additionally, they inhibit cell metastasis and activate immune cells, thereby enhancing the host’s immune system through indirect stimulation of humoral and cell-mediated immunity to suppress tumors. They also directly induce apoptosis in tumor cells [55]. In traditional Chinese medicine, *Sanghuangporus* is primarily utilized for alleviating allergies, enhancing blood circulation, safeguarding liver health, alleviating gastrointestinal ailments, and playing a crucial role in combating tumors. Examples of the preparation of *Sanghuangporus* polysaccharides have been available since the mid-20th century. Studies have demonstrated that these polysaccharides possess potent antitumor and immunostimulatory activities in cancer cell lines. They regulate oxidative stress and influence signaling pathways such as NF-κB, KEGG, MAPK, and PI3K/AKT, thereby improving chronic diseases. This provides evidence of the antitumor properties of the polysaccharide components in *Sanghuangporus*. *Sanghuangporus* polysaccharides have been documented to enhance T lymphocyte proliferation and humoral immune function. They act as a polyclonal activator of B cells, induce secretory and cellular macrophage responses, and suppress tumor growth and metastasis through immune enhancement. Su showed that *Sanghuangporus* polysaccharides exert antitumor effects via the P53 signaling pathway [56]. An essential tumor suppressor, they play crucial roles in regulating cell cycle, apoptosis, and cell senescence, among other processes. By mediating apoptosis and cell cycle, they effectively hinder tumor proliferation, thus serving a beneficial role in suppressing malignant tumor growth. *Sanghuangporus* polysaccharides exhibit potent antitumor effects by stimulating T cells to modulate gut microbiota and enhance intestinal metabolism. Therefore, targeting the intestinal microbiota could emerge as a novel approach for treating colorectal cancer. The anticancer properties of certain fungal bioactive compounds have been identified and examined for their efficacy against cancer. Building on this foundation, there is significant research value in advancing the development and exploration of new potential anticancer medications, particularly natural compounds. These compounds not only hold promise for tumor treatment but also have the potential to directly trigger tumor cell apoptosis with minimal side effects. Currently, research into the antitumor mechanisms of polysaccharides from *Sanghuangporus* is relatively abundant. Hence, investigating the mechanisms of polysaccharide components in *Sanghuangporus* holds significant importance in impacting tumor development. Figure 4 illustrates the antitumor mechanism of *Sanghuangporus* polysaccharides.

### 4.2. Inhibitory Effect of Sanghuangporus Polysaccharides on Gastric Cancer

Gastric cancer, a common malignancy affecting the gastrointestinal tract, arises from genetic mutations in human cells. Natural plants are commonly employed in contemporary medicine for tumor prevention or treatment purposes. Polysaccharides extracted from natural plants can influence gastric cancer by modulating several signaling pathways, including the PI3K/AKT, MAPK, Fas/FasL, Wnt/β-catenin, IGF-IR, and TGF-β pathways. Additionally, these polysaccharides can trigger apoptosis in gastric cancer cells, halt the cell cycle, and suppress migration and invasion. Moreover, it was discovered that the polysaccharides induce apoptosis in SGC-7901 cells by blocking the AKT/PI3K signaling pathway. This was accomplished by upregulating the expression of caspase-8, caspase-3, p53, Bax, and Bad while downregulating the expression of Bcl-2 and Bcl-xl through inhibition of the PI3K/AKT signaling pathway. Additionally, the polysaccharides were noted to reduce the expression of MMP-2, MMP-9, Snail, and waveform protein, while increasing E-cadherin expression [57]. Polysaccharides derived from *Sanghuangporus* can suppress tumor growth and metastasis while also stimulating immune cells like T cells, B cells, natural killer cells, and macrophages. These immune cells possess the capability to eradicate cancer cells and pathogens. Recent research indicates that the antitumor mechanisms of *Sanghuangporus* involve the induction of apoptosis, secretion, and macrophage response. As a result, *Sanghuangporus* has garnered considerable interest due to its strong antitumor properties. Previous studies have demonstrated that *Sanghuangporus* polysaccharides can boost immune function and stimulate T lymphocyte proliferation. Furthermore, the administration of 100 mg/kg of *Sanghuangporus* polysaccharide markedly enhanced the survival rate of B16F10-transplanted mice, inhibited tumor growth, decreased tumor weight, and reduced the occurrence of lung metastasis in B16F10 melanoma. The research examined the anticancer effects of *Sanghuangporus* polysaccharides on human melanoma and gastric adenocarcinoma. The findings revealed an elevated apoptosis rate in both types of cancer cells, reduced tumor weight, and prolonged survival of the mice. The research indicates that *Sanghuangporus* polysaccharides could serve as a promising treatment option for melanoma and gastric adenocarcinoma [58]. Ming-Ming Liu conducted a cell proliferation assay using the gastric cancer cell line SGC-7901. The findings revealed that *Sanghuangporus* polysaccharide treatment significantly suppressed the growth of SGC-7901 cells. Furthermore, the cells’ morphology transitioned from elongated to oval shape. Flow cytometry analysis indicated that the administration of *Sanghuangporus* polysaccharides did not cause a significant change in the proportion of cells in the G2/M phase. However, there was a notable reduction in the percentage of cells in the S phase, coupled with a substantial rise in the percentage of cells in the G0/G1 phase, increasing from 56.08% to 62.84%. These findings indicate that *Sanghuangporus* polysaccharides hinder the proliferation of SGC-790 cells. Jia-He Wang employed atomic force microscopy to assess alterations in parameters including the morphology, surface roughness, and adhesion force of gastric cancer cells pre and post exposure to *Sanghuangporus*. The findings indicated that *Sanghuangporus* exerted a substantial cytotoxic impact on SGC-7901 gastric cancer cells, causing a transition in cell morphology from flat to spherical. This transition was accompanied by heightened height and surface roughness values, as well as diminished adhesion force. Research has revealed that *Sanghuangporus* can enhance the body’s immune function and suppress the proliferation of gastric cancer cells, leading to increased resistance against gastric cancer cells [59]. Jae-Sung Bae investigated the impact of polysaccharides extracted from *Sanghuangporus* (PG) on antral gastric cancer induced by benzo(a)pyrene (BaP) in mice. The research observed that mice administered *Sanghuangporus* polysaccharides for four weeks showed minimal hyperplasia and no papillomas. This indicates that polysaccharides extracted from PG might hinder BaP-induced mouse antral carcinogenesis by decreasing the expression of mutant p53 [31].

### 4.3. Inhibitory Effect of Sanghuangporus Polysaccharides on Liver Cancer

The liver, as the largest organ in the body, plays significant roles in protein synthesis, metabolism, detoxification, and the maintenance of homeostasis. Various severe liver conditions exist, such as hepatitis, fibrosis, hereditary and metabolic disorders, and hepatocellular carcinoma, which ranks among the primary causes of cancer-related fatalities globally. The primary form of liver cancer is mainly hepatocellular carcinoma (HCC), which may arise due to viral hepatitis, alcohol abuse, and metabolic disorders. Hepatocellular carcinoma (HCC) involves numerous molecular pathways and can develop in a liver that is afflicted with disease. Additionally, primary liver cancers encompass cholangiocarcinoma, fibrolamellar carcinoma, hepatoblastoma, angiosarcoma, and various other interstitial carcinomas of the liver. Secondary liver cancers comprise metastatic tumors originating from breast, lung, pancreatic, and colorectal cancers [60]. This review compiles current research on *Sanghuangporus’* potential in treating hepatocellular carcinoma and offers theoretical insights for its future advancement. Guibin Wang employed RT-PCR to measure the levels of Bcl-2 and Bax mRNA. The findings indicated a notable decrease in Bcl-2 mRNA expression with rising concentrations of *Sanghuangporus* polysaccharide, while there was a significant increase in Bax mRNA expression as the concentration of *Sanghuangporus* polysaccharide increased. The results indicate that *Sanghuangporus* polysaccharide treatment modulated the expression of Bcl-2 and Bax genes in HepG2 cells. This indicates that PL inhibits the growth of HepG2 cells by inducing a blockage in the S phase of the cell cycle. Additionally, PL decreased tumor cell adhesion and invasiveness in a manner dependent on both dose and time. You-Gui Li’s team investigated how proteoglycan P1 from Phellinus linteus (Mesima) affects human hepatocellular carcinoma cells (HepG2) [61]. They conducted both in vivo and in vitro experiments to observe how P1 affects the proliferation of HepG2 cells. The findings indicated a significant reduction in tumor size in HepG2 cells treated with P1 compared to the control group, with tumors being notably reduced. Compared to the control group, HepG2 cells treated with P1 exhibited a significant dose-dependent increase in the S-phase block. In P1-treated HepG2 cells, the expression of calreticulin, cyclin D1, cyclin E, and CDK2 was significantly decreased, whereas the expression of P27kip1 and cyclin A was increased. These findings imply that calreticulin expression and the P27kip1, cyclin A/D1/E, and CDK2 pathways play a role in the S-phase cell cycle arrest induced by P1 in HepG2 cells. *Sanghuangporus* proteoglycan P1 inhibits the growth of human HepG2 cells by inducing a blockade in the S phase of the cell cycle. This was accomplished by activating the P27kip1, cyclin A/D1/E, and CDK2 pathway and enhancing calreticulin expression. Furthermore, Zhao Miaohui explored the mechanism by which *Sanghuangporus* polysaccharides inhibit tumor growth. This was accomplished under a mouse model, quantifying the levels of VEGF, IL-1β, TNF-α, and IL-6 in the tumors via ELISA, and evaluating the expression of mTOR, p-mTOR, AKT, p-AKT, and PI3K.

Western blot analysis was employed to examine the expression of the p-PI3K protein in mouse tumor tissues. The study aimed to investigate how *Sanghuangporus* polysaccharides enhance the effectiveness of chemotherapy and reduce toxicity in mice with hepatocellular carcinoma ascites by modulating the PI3K/AKT/mTOR pathway. The results showed that *Sanghuangporus* polysaccharides significantly increased the rate of tumor suppression, decreased tumor volume, altered the inflammatory environment within tumor tissues, and restrained tumor growth in mice, demonstrating inhibitory effects on hepatocellular carcinoma.

### 4.4. Inhibitory Effect of Sanghuangporus Polysaccharide on Colorectal Cancer

Colorectal cancer (CRC) is the third most prevalent cancer in both men and women. The etiology of colon cancer involves the Wnt/β-catenin signal. The development of colon cancer is closely associated with the Wnt/β-catenin signaling pathway. *Sanghuangporus* polysaccharides suppress tumor growth, invasion, and angiogenesis by inhibiting Wnt/β-catenin signaling in specific colon cancer cells. Extensive research has been conducted on the inhibitory effects of *Sanghuangporus* polysaccharides on colon cancer. PL has been discovered to markedly decrease β-catenin protein levels and to downregulate genes associated with the Wnt/β-catenin pathway, leading to decreased invasiveness of SW480 colon cancer cells. The data available suggest that the downregulation of Wnt/β-catenin signaling induced by PL may play a role in inhibiting the growth, invasion, and angiogenesis of SW480 colon cancer cells. Both in vitro and in vivo experiments have shown that PL notably decreased β-catenin levels and downregulated specific downstream genes within the Wnt/β-catenin pathway in SW480 colon cancer cells. Moreover, PL decreased the invasiveness of SW480 cells by directly impacting the activity, motility, and angiogenesis of matrix metalloproteinases (MMPs), which are intricately linked to Wnt/β-catenin signaling. Furthermore, protein-bound polysaccharides extracted from *Sanghuangporus* triggered G2/M phase arrest and apoptosis in human colon cancer SW480 cells by suppressing Wnt/β-catenin signaling [3]. Therefore, PL suppresses the Wnt/β-catenin signaling pathway and is a potential therapeutic agent for individuals with colon cancer. As a result, PL inhibits the Wnt/β-catenin signaling pathway and has the potential to be developed into an effective treatment for colon cancer patients. Shanshan Guo utilized water, 60% ethanol, and 95% ethanol to extract *Sanghuangporus*. Among these extracts, it was observed that the 60% ethanolic extract of *Sanghuangporus* effectively suppressed the proliferation of SW480 colon cancer cells and decreased the activation of the AKT/mTOR signaling pathway [62].

Furthermore, it notably suppressed gene expression and exhibited enhanced anticancer efficacy, potentially slowing the development of colorectal cancer. Tsuji found that *Sanghuangporus* polysaccharides decreased cancer cell viability by reducing the protein expression of Bcl-2 and cyclin B1 [63]. This was accomplished by activating caspase 2, caspase 8, and apoptotic pathways related to endoplasmic reticulum stress. Hye-Jin Park conducted a study on *Sanghuangporus* extract (PBR) cultivated on germinated brown rice. The research demonstrated that PBR increased the expression of p21CIP1/WAF1 via the p53 pathway. This resulted in reduced cyclin D1 expression and a notable, dose-dependent rise in p21CIP1/WAF1 protein expression. The study revealed that PBR disrupts the cell cycle by controlling the expression of regulatory proteins involved in the G1 phase. Moreover, PBR notably decreases the expression of Bcl-2 and suppresses the proliferation of colon cancer cells in a manner dependent on both time and dosage. These results indicate that PBR could be a promising treatment option for colon cancer [64]. It has been noted that the antitumor efficacy of polysaccharides rises with their molecular weight. Yuxia Mei analyzed the structures of two polysaccharides, PLPS-1 and PLPS-2, isolated from Morus alba. The results showed that the backbone of PLPS-1 mainly consisted of repeating α-d-Glc(1 → 4)-α-d-Glc (1→6) units, whereas that of PLPS-2 contained α-(1 → 3)-d-Glc and α-(1 → 6)-d-Glc. The two branches of polysaccharides exhibited distinct carbohydrate compositions. It is hypothesized that the notable difference in antitumor activity between the two PLPSs is attributed to these structural differences. Prior studies have shown that PL’s novel proteoglycan (P1) inhibits colorectal cancer by boosting the immune response to T cells and IgA, and by disrupting the Reg IV/EGFR/Akt signaling pathway. P1 triggers apoptosis and halts the cell cycle in the G2/M phase in SW480 cells by decreasing the levels of Bcl-2 and cell cycle protein B1 expression. *Sanghuangporus* polysaccharides inhibit the growth of human colorectal cancer cells by stimulating their P27kip1-cyclin D1/E-CDK2 pathway, leading to the arrest of the cell cycle in the S phase [65]. Shi Zhong examined the impact of *Sanghuangporus* polysaccharides on cell proliferation, cell cycle distribution, apoptosis, autophagy, and the expression of various cell cycle-related proteins in HT-29 cells both in vitro and in vivo, aiming to acquire a more comprehensive understanding of their mode of action. Proteoglycans extracted from *Sanghuangporus* were observed to inhibit the growth of colorectal cancer HT-29 cells, both in vitro and in vivo. This inhibition was achieved by slowing down the cell cycle in the S phase and reducing the levels of cyclin D1, cyclin E, and CDK2 while upregulating the expression of P27kip1. The findings from RT-PCR and Western blot analysis show a notable downregulation in the expression of cyclin D1, cyclin E, and CDK2, along with an upregulation in P27kip1 levels in HT-29 cells treated with P1 at a concentration of 100 μg/mL. These results imply that P27kip1-cyclin D1/E-CDK2 pathway activation plays a role in the P1-induced blockage of the S-phase cell cycle in HT-29 cells. You-Gui Li isolated a novel heteropolysaccharide named P1 from Morus alba and examined its anticancer properties. The results evaluation revealed that P1 can directly suppress colorectal cancer by disrupting the signaling pathway involving Reg IV/EGFR/Akt [66]. Experimental findings demonstrate that P1 suppresses the proliferation of HT-29 cells by reducing the expression of Reg IV and EGFR genes and increasing pIgR mRNA levels. Moreover, P1 boosts T cell and IgA immune responses and concurrently inhibits colorectal cancer by disrupting the Reg IV/EGFR/Akt signaling pathway. Therefore, *Sanghuangporus* polysaccharides exhibit not only immunomodulatory effects but also possess the capability to directly target tumor cells, thereby positioning them as a promising candidate for antitumor medication.

### 4.5. The Inhibitory Effect of Sanghuangporus on Breast Cancer

Breast cancer (BC) ranks as the second most prevalent cause of female mortality globally. Its onset is attributed to a range of factors such as physical activity, age, breast tissue density, familial background, and genetic mutations. Approximately 25% of hereditary BC has been demonstrated to result from mutations of BC susceptibility gene-1 (BRCA1), which increase the incidence of early-onset BC up to 80%. Nanomedicine, immunotherapy, and stem cell transplantation have been employed in the management of breast cancer. In addition to human epidermal growth factor receptor-2 (HER2), epidermal growth factor receptor (EGFR), or human epidermal growth factor receptor-1 (HER1), is another oncogene in the ErbB family. Together with the over expression of mouse double minute 2 (MDM2), it has a role in BC via the augmentation of BC invasion and migration through negatively regulating P53 as well as tumor necrosis factor α (TNFα), which elicits inhibition of cell proliferation, induction of apoptosis, and even the enhancement of cell migration, as well as contributing to poor prognosis outcomes. The co-existence between PI3KCA, a catalytic subunit of the PI3K protein, with the Kirsten ras oncogene (KRAS) mutation was identified in advanced-grade carcinogenesis, together with KRAS gene silencing, which resulted in inactivation of the PI3KCA pathway; PI3KCA and KRAS are therefore good candidate genes to study. Because herbs contain diverse bioactive compounds and exhibit various pharmacological effects, *Sanghuangporus* has been shown to have substantial antitumor properties. Polysaccharides, specifically β-glucans, are the main active constituents responsible for influencing both antiproliferative effects and immune response modulation. Kanwalat Chalertpet found that *Sanghuangporus* reduced the viability and proliferation of breast cancer cell lines in a manner dependent on both dosage and duration. The proportion of cells in the S phase was notably diminished, and it could also impact the G0/G1 cell cycle block. D Sliva examined how PL affects highly invasive and metastatic human breast cancer cells. The findings indicated that PL inhibits the proliferation and formation of colonies in these cells. Moreover, PL restrained the invasive tendencies of MDA-MB-231 cells by reducing cell adhesion, migration, and invasion. The colony-forming ability of MDA-MB-231 cells was evaluated, revealing their formation of colonies on agar following a 14-day incubation period. Higher concentrations of PL led to a notable reduction in the number of colonies formed. Thus, *Sanghuangporus* suppressed cell proliferation and the formation of colonies in highly invasive breast cancer cells. Cell cycle analysis showed that PL induced cell cycle arrest in the S phase and markedly upregulated the expression of p27, an inhibitor of cell cycle protein-dependent kinase. The increased expression of p27 suppressed the growth of breast cancer cells, leading to arrest in the S phase of the cell cycle and the inhibition of tumor growth. The research demonstrated that PL hinders the proliferation and colony formation of highly aggressive breast cancer cells by triggering S-phase cell cycle arrest through the upregulation of p27 expression. Moreover, the arrest of the cell cycle in the S phase could also be seen as a halt in the G1/S phase, considering that the majority of cells are in the G0/G1 and S phases, but not in the G2 phase (Table 1). Notably, PL suppresses the growth of cancer cells by causing cell cycle arrest.

### 4.6. The Inhibitory Effect of Sanghuangporus on Prostate Cancer

Alterations in the androgen receptor (AR) have been shown to play a role in prostate cancer development. Studies by Tsuji’s group have shown that *Sanguangporus* can suppress tumor growth, invasiveness, and angiogenesis in the SW480 tumor xenograft model in nude mice by blocking the Wnt/β-catenin signaling pathway, inducing apoptosis and cancer regression, and slowing prostate cancer growth. Experimental data showed that a polysaccharide-rich extract of *Sanguangporus* effectively suppresses the proliferation of prostate cancer cell lines. Nude mice were used in the experiments, and after administration of *Sanguangporus* polysaccharide extract to nude mice, tumors induced by inoculation of DU145 or PC3 cells regressed due to the triggering of the apoptotic pathway. This confirmed the ability of PL to induce apoptosis in tumor cells. T. Zhou showed that the combination of low-dose PL with the anticancer drug doxorubicin synergistically induced apoptosis in LNCaP prostate cancer cells. High-dose PL was found to activate two important apoptotic pathways in prostate cancer cells: caspase 8-induced cascade response and unfolded protein response. High doses of PL have been shown to arrest prostate cells in the G1 phase of the cell cycle by inhibiting the expression of cell cycle protein D1 and its interaction with CDK4 and CDK6. In normal prostate epithelial cells, PL treatment can control the G1 checkpoint, resulting in cell cycle arrest. The dispersion of polysaccharides induces apoptosis and initiates prostate cancer cell death, thereby inhibiting prostate cancer growth. This effect is particularly important for cancer cells, which often have cell cycle arrest points. The polysaccharide *Sanguangporus* is known for its strong antitumor properties. It is able to inhibit gastric, liver, colon, breast, and prostate cancers by targeting various signaling pathways, such as Wnt, β-catenin, Reg IV, EGFR, and Akt.

Reducing the expression of these related genes results in a marked reduction in tumor cell proliferation. Therefore, *Sanghuangporus* polysaccharides show promise as effective therapeutic agents for certain types of tumors and have favorable prospects for further development.

## 5. Summary and Prospect

In recent times, polysaccharides, phenols, triterpenoids, and other constituents derived from *Sanghuangporus* have garnered significant attention from researchers due to their beneficial biological properties. Among these, polysaccharides isolated and purified from the fruiting bodies, mycelium, and fermentation broth of *Sanghuangporus* are recognized as the primary bioactive components. These polysaccharides exhibit valuable effects, including immunomodulation, antitumor activity, anti-inflammatory properties, hypoglycemic effects, and hypolipidemic effects. This review has explored three key dimensions of *Sanghuangporus* polysaccharides: extraction methods, structural properties, and antitumor mechanisms. A variety of extraction methods for *Sanghuangporus* polysaccharides have been presented, including hot water extraction, enzyme-assisted extraction, ultrasonic-assisted extraction, microwave extraction, and ultrasonic–microwave-assisted extraction. The advantages and disadvantages of each method have been carefully deliberated, along with their current applications. These data will assist readers in choosing the most efficient and effective approach for extracting polysaccharide components from *Sanghuangporus*. Additionally, this research offers a comprehensive structural analysis of *Sanghuangporus* polysaccharides. The research encompasses data on molecular weight, monosaccharide composition, glycosidic bond type, and spatial conformation. Lastly, the antitumor mechanism of *Sanghuangporus* polysaccharides was investigated. It was revealed that *Sanghuangporus* polysaccharides exhibited a notable inhibitory effect on malignant tumors, including gastric, hepatic, colorectal, breast, and prostate cancers. This finding holds significant implications for the food, cosmetic, and pharmaceutical sectors. With the growing focus on health and rising demand for natural remedies, *Sanghuangporus* polysaccharide, derived from a natural plant extract, holds vast potential for diverse applications. Given its array of biological activities, including antioxidant, anti-inflammatory, and immunomodulatory effects, *Sanghuangporus* polysaccharide is anticipated to play a crucial role in the prevention and treatment of inflammatory diseases and tumors. The natural, safe, and non-toxic properties of *Sanghuangporus* polysaccharides make them an excellent substitute for chemical medications. Despite the significant biological activity demonstrated by *Sanghuangporus* polysaccharides both in laboratory settings and in living organisms, particularly in terms of their antitumor effects, research in this area remains limited. Future studies should prioritize investigating the correlation between the structure of *Sanghuangporus* polysaccharides and their antitumor properties. Furthermore, further research is required to elucidate the antitumor mechanisms of *Sanghuangporus* polysaccharides at both the cellular and molecular levels. Enhanced characterization and a deeper understanding of the signaling pathways influenced by these fungal polysaccharides will facilitate the development of innovative medications, cosmetics, and functional foods.

## Data Availability

The original contributions presented in the study are included in the article, further inquiries can be directed to the corresponding author.
